# Atorvastatin-mediated downregulation of VCAM-1 and XO/UA/caspase 3 signaling averts oxidative damage and apoptosis induced by ovarian ischaemia/reperfusion injury

**DOI:** 10.1080/13510002.2022.2129192

**Published:** 2022-10-06

**Authors:** O. A. Afolabi, M. A. Hamed, D. C. Anyogu, D. H. Adeyemi, A. F. Odetayo, R. E. Akhigbe

**Affiliations:** a Department of Physiology, Ladoke Akintola University of Technology, Ogbomoso, Nigeria; b Brainwill Laboratories, Osogbo, Nigeria; c Reproductive Biology and Toxicology Research Laboratory, Oasis of Grace Hospital, Osogbo, Nigeria; d Department of Veterinary Pathology and Microbiology, University of Nigeria, Nsukka, Nigeria; e Department of Physiology, Faculty of Basic Medical Sciences, College of Health Sciences, Osun State University, Osogbo, Nigeria; f Department of Physiology, University of Ilorin, Ilorin, Nigeria

**Keywords:** Adhesion molecules, folliculogenesis, inflammation‌, ischaemia/reperfusion, ‌oocyte, ovary, statins‌, torsion/detorsion

## Abstract

**Background:**

Oxidative damage is critical in the pathogenesis of ovarian ischaemia/reperfusion (I/R) injury, and statins have been reported to exert antioxidant activity. However, the role of VCAM-1 and xanthine oxidase (XO)/uric acid (UA) in ovarian I/R injury is not known. Also, whether or not atorvastatin exerts antioxidant activity like other statins is unclear.

**Objectives:**

This study investigated the involvement of VCAM-1 and XO/UA in ovarian I/R injury and the likely protective role of atorvastatin.

**Methods:**

Forty female Wistar rats were randomized into sham-operated, ischaemia, ischaemia/reperfusion (I/R), ischaemia and atorvastatin, and I/R and atorvastatin.

**Results:**

In comparison with the sham-operated group, atorvastatin blunted ischaemia and I/R-induced distortion of ovarian histoarchitecture and follicular degeneration. Also, atorvastatin alleviated ischaemia and I/R-induced rise in XO, UA, and malondialdehyde, which was accompanied by inhibition of ischaemia and I/R-induced reductions in reduced glutathione level, enzymatic antioxidant activities and increase in myeloperoxidase activity and TNF-α and IL-6 levels by atorvastatin treatment. Additionally, atorvastatin blocked ischaemia and I/R-induced increase in VCAM-1 expression, caspase 3 activity, 8-hydroxydeoxyguanosine level and ovarian DNA fragmentation index.

**Conclusion:**

For the first time, this study revealed that atorvastatin-mediated downregulation of VCAM-1 and XO/UA/caspase 3 signaling averts oxidative injury, inflammation, and apoptosis induced by ovarian ischaemia/reperfusion injury.

## Introduction

Ischaemia/reperfusion (I/R) injury is a rare but severe cause of multiple organ damage. The ovaries [1], just like the brain [2], heart [3], liver [4], kidney [5], and testes [6] have been documented to suffer I/R injury. Although the incidence rates vary, ovarian torsion also known as adnexal torsion or tubo-ovarian torsion, affects both children and adolescents [7, 8]. This gynaecological emergency has been reported to have a prevalence of 2.7–7.4% among women in their reproductive ages [7], and about 15% among infants and children [8]. Early diagnosis and management increases the chance of preservation of ovarian function and fertility [9].

Although the mechanism associated with cellular injury in ovarian torsion is yet to be fully elucidated, the main aetiopathophysiologic event has been reported to be ischaemia preceding reperfusion [10]. Ischaemia impairs ovarian blood flow with attendant vascular congestion, hemorrhage and necrosis [11], while detorsion following ovarian torsion promotes neutrophil infiltration and excessive production of reactive oxygen species (ROS) like superoxide radical, hydrogen peroxide, hydroxyl radical, and nitric oxide [12]. The generated ROS trigger lipid peroxidation (with maldondialdehyde, MDA, generated as the end product) and DNA damage (with 8-hydroxyguanine, 8OHdG, generated as the end product) in the cellular and mitochondrial membranes [12, 13]. Cells and tissues protect themselves from oxidative injury by the buffering capacity of enzymatic and non-enzymatic antioxidants [14], notwithstanding, the excessive production of ROS in I/R causes a redox imbalance in favor of oxidative stress that results in lipid peroxidation, protein denaturation, and oxidative DNA damage [12].

Furthermore, vascular cell adhesion molecule (VCAM-1), a cytokine-inducible adhesion molecule, has been reported to play a key role in I/R injury in testis [15] and liver [16]. Also, modulation of xanthine oxidase (XO)/uric acid (UA) signaling has been reported to influence I/R-induced testicular injury [6]. Despite the studies on ovarian torsion, little or none is known about the role of VCAM-1 and XO/UA/caspase 3 signaling in the pathogenesis of I/R injury in ovarian torsion.

An increasing number of studies have emerged on the beneficial effects of antioxidant and anti-inflammatory agents on I/R-induced injuries [17, 18]. Also, the off-label benefits of known therapeutic molecules such as metformin [19], amlodipine [10], nimodipine [20], and losartan [11] on ovarian torsion/detorsion (T/D)-induced I/R injuries have been reported. However, data on the effect on atorvastatin, a statin, on I/R injury in ovarian torsion is scarce. Statins are hydroxymethylglutaryl-coenzyme A (HMG-CoA) reductase inhibitors that are known for their lipid-lowering effects. Convincing shreds of evidence have demonstrated that some statins exert anti-inflammatory and antioxidant activities via modulation of adhesion molecules such as VCAM-1 [21, 22], though there is a dearth of information in the literature on the effect of atorvastatin on VCAM-1 [21–23].

Given the afore-mentioned information, it was hypothesized that VCAM-1 and XO/UA/caspase 3 signaling are key players in torsion/detorsion-driven I/R-induced ovarian injury. Also, it was hypothesized that atorvastatin would suppress VCAM-1. Hence, the present study assessed the impact of atorvastatin on VCAM-1 and XO/UA/caspase 3 signaling, as a possible mechanistic pathway, in I/R-induced ovarian injury.

## Materials and methods

### Animals and treatments

The study was approved by the Ethics Committee of the Faculty of Basic Medical Sciences, Ladoke Akintola University, Ogbomoso, Nigeria and carried out in accordance with the guidelines of the 'National Institute of Health using the guide for the care and handling of laboratory animals (NIH Publication No. 80-23; revised 1978)'. Forty female Wistar rats were obtained from the animal holdings of the Department of Physiology, Ladoke Akintola Universiy of Technology, Ogbomoso, Nigeria. Animals were acclimatized for two weeks and then randomized into five groups (*n *= 8): the sham-operated (sham), ischaemia (I), ischaemia/reperfusion (I/R), ischaemia + atorvastatin (I + AT), and ischaemia/reperfusion + atorvastatin (I/R + AT). The sham-operated rats received 0.5 ml of distilled water, while the I + AT and I/R + AT received 5 mg/kg of AT in addition to undergoing torsion and torsion/detorsion respectively. The animals in the I and I/R groups underwent torsion and torsion/detorsion only respectively. Torsion lasted for three hours before detorsion. After detorsion, reperfusion was allowed for another three hours before termination of the study. Atorvastatin was administered via gavage two hours after the induction of torsion. The timeline of torsion, torsion/detorsion, and atorvastatin administration was to mimic the real-time experience in humans. The dose of atorvastatin used in this study is the mean human equivalent dose of 10–80 mg daily. The animals were nulliparous and the oestrous cycle of all animals was assessed daily by vaginal smear technique using 0.1% Crystal violet stain and viewed by light microscopy [24]. Animals were monitored for three consecutive cycles, and only those with a regular cycle and at the same phase of cycle were selected for the study [25].

Bilateral ovarian T/D was carried out to surgically induce I/R as previously reported [19, 20]. Briefly, animals were anaesthetized with ketamine (40 mg/kg) and xylazine (4 mg/kg) *i.p*. The fur over the incision site was shaved and cleaned with chlorhexidine solution, after which a 2-cm longitudinal incision was made in the midline area of the lower abdomen and developed into the pelvic cavity until the uterine horns and adnexa were reached. The ovarian pedicles were twisted 720° clockwise around its axis and clamped with atraumatic vascular clips applied about 1 cm above and below the ovary to induce ischaemia for three hours. The skin was closed with 3/0 silk suture and covered with sterile gauze pad for three hours, then detorsion was carried out by removing the clamps and untwisting the ovarian pedicles to allow reperfusion. The skin was apposed with vicryl suture, and 10% Povidone-iodine applied. The animals in the sham-operated group underwent a similar procedure but the ovarian pedicles were neither twisted nor clamped. Procedure was carried out under aseptic conditions after which all animals received subcutaneous ciprofloxacin 0.1 mg/kg as prophylaxis against infection. Animals were returned to their respective cages after the surgical procedures and left for three hours before terminating the study.

### Sample preparation

At the end of the experiment, animals were euthanized and the ovaries were excised and separated from adhering structures. The right ovaries were put in appropriate volume of cold phosphate buffer solution and stored at −20°C for biochemical assays, while the left ovaries were fixed in 10% neutral buffered formalin for histopathological examination.

### Biochemical assays

About 0.1 g of the right ovaries of each animal were homogenized on ice in 10 ml of cold phosphate buffer solution. The resultant solutions were centrifuged at 10,000 rpm for15 mins in cold centrifuge after which the supernatant was decanted into tubes and stored at −20°C for biochemical assay later.

The ovarian concentrations of UA [26], MDA [27], and GSH [28] were assayed by colorimetric method as previously reported. The activities of XO [27], superoxide dismutase (SOD) [28], catalase [28], glutathione peroxidase (GPx) [29], glutathione-S-transferase (GST) [27], and myeloperoxidase (MPO) [29] in the ovarian tissues were assayed by colorimetry as earlier documented. Also, the ovarian levels of TNF-α (Elabscience Biotechnology Inc., U.S.A.), IL-6 (Elabscience Biotechnology Inc., U.S.A.), 8OHdG (Elabscience Biotechnology Inc., U.S.A.), and VCAM-1 (Elabscience, Wuhan, China) as well as the activity of caspase 3 (Elabscience Biotechnology Inc., U.S.A.) were determined using ELISA kit following the manufacturers’ guidelines. Ovarian DNA fragmentation index was determined by diphenylamine methods as previously reported [25].

### Histopathological analysis

Ovarian histopathological analysis was carried out as earlier reported [25]. Briefly, the fixed ovarian tissue was dehydrated and embedded in paraffin wax. About 4 µm thick sections were stained with haematoxylin and eosin, and examined under light microscope. Photomicrographs were taken at 100x magnification using a computerized digital camera attached to the microscope (Omax Digital Microscope, China).

Ovarian histoarchitecture was scored as previously reported [25, 30]. The absence or presence of about <33%, 33–66%, and >66% of follicular degeneration, vascular congestion, haemorrhage, oedema, and infiltration by inflammatory cells were scored as 0, 1, 2, and 3 respectively. Follicles were classified as 'atretic follicles' if two or more of these criteria are met: 'more than two pyknotic nuclei in the granulosa cell layer, cell debris within antral cavity, granulosa cells pulling away from the basement membrane, swelling in the theca cells, degenerating oocyte and degenerated zona pellucida'. Five replicates per group were independently scored by two experts who were blinded to the study protocol. Five fields were viewed per section, and the average score was recorded as the score of the variable for that slide. Statistical analysis.

Values are reported as mean ± standard deviation. Statistical analysis was conducted with Graph Pad Prism (Versions 5.01, GraphPad Software, Inc.). One-way analysis of variance (ANOVA) was employed to compare the mean values of variables across the groups, while Tukey’s posthoc test was used to identify the significance of pair wise comparisons of the mean values of the groups. Results were considered statistically significant at *p* < 0.05.

## Results

### Histopathological evaluation

**Figure 1.**
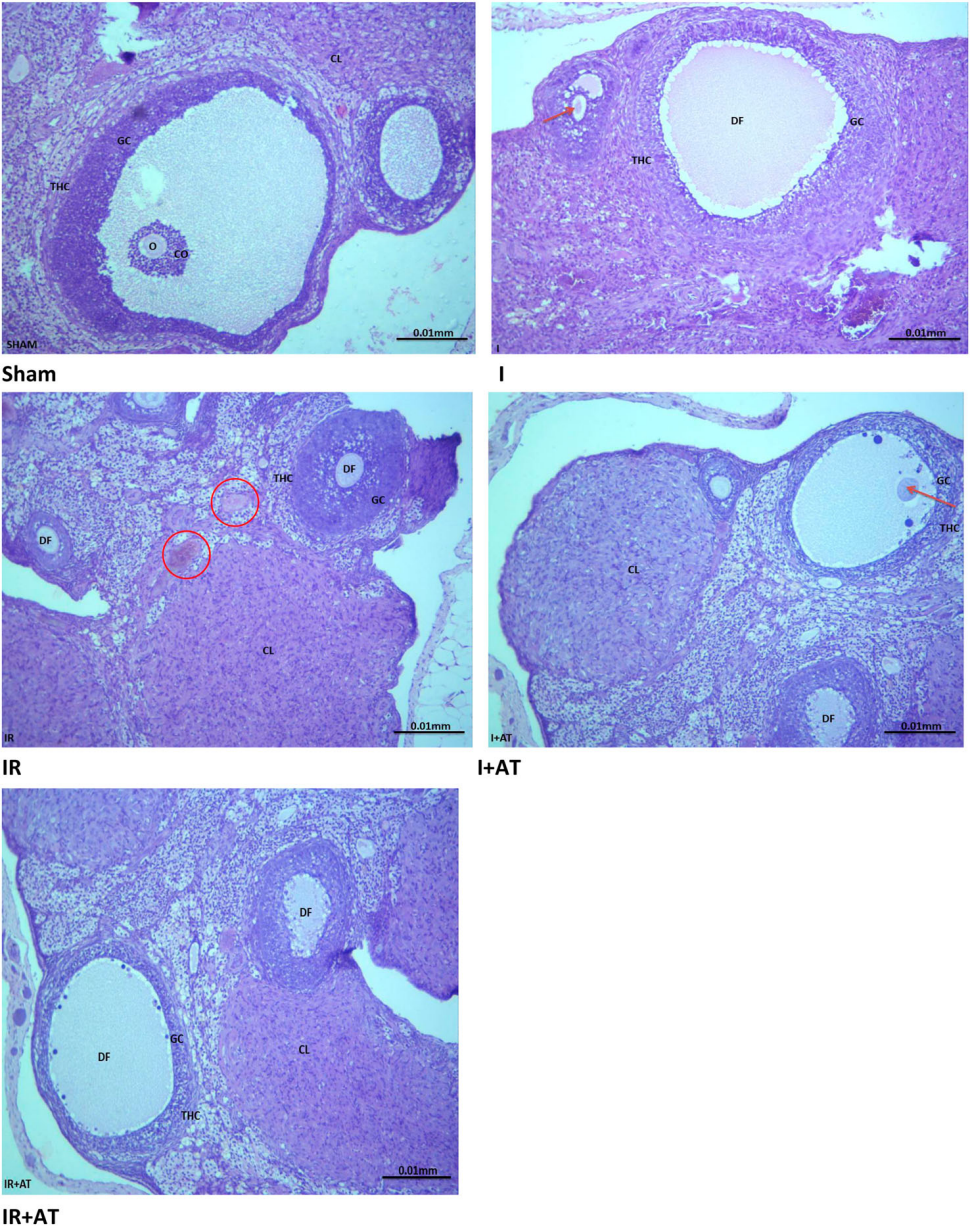
Photomicrographs of the ovarian tissues. Photomicrographs are representatives of 5 replicates per group. The sham-operated rats showed preserved ovarian histoarchitecture. There were oocytes (O) surrounded by cumulus oophorus (CO) that were lined by granulosa cells (GC) and theca cells (THC). The corpus luteum appeared normal. The rats in the ischaemia group (I) showed moderately distorted ovarian histoarchitecture. There were some degenerated follicles (DF) and some developing ooctes (red arrow). The granulosa cell (GC) and theca cells (THC) appeared normal. The rats in the ischaemia/reperfusion group (IR) showed grossly distorted ovarian histoarchitecture. There were degenerated follicles (DF) with distorted granulosa cell (GC) and theca cells (THC) that appeared infiltrated by inflammatory cells. The instertitium appeared congested (red circle). The animals in the ischaemia + atorvastatin group (I + AT) showed mildly distorted ovarian histoarchitecture. There were some degenerated follicles (DF) and some developing follicles (red arrow). The granulosa cell (GC) and theca cells (THC) appeared normal. Also, the corpus luteum (CL) appeared normal. The animals in the ischaemia/reperfusion + atorvastatin group (IR + AT) showed moderately distorted ovarian histoarchitecture. There were degenerated follicles (DF) with normal granulosa cell (GC) and theca cells (THC). The corpus luteum (CL) appeared normal.

The sham-operated rats showed normal ovarian histoarchitecture, while those in the ischaemia group exhibited moderate distortion in the ovarian histoarchitecture with some degenerated follicles. The rats in the ischaemia/reperfusion group showed grossly distorted ovarian histoarchitecture with degenerated follicles, distorted granulosa and theca cells, and congested interstitium. In addition, the rats in the ischaemia + atorvastatin group showed mild distortion of the ovarian histoarchitecture with some degenerated and developing follicles, while those in the ischaemia/reperfusion + atorvastatin group showed moderately distorted ovarian histoarchitecture with degenerated follicles (Figure 1).

There was a significant increase in follicular degeneration, vascular congestion, oedema, haemorrhage, and infiltration by inflammatory cells following ischaemia compared with the sham-operated. The observed ischaemia-induced histopathological lesions were ameliorated by atorvastatin. Also, ischaemia/reperfusion led to a significant increase in follicular degeneration, vascular congestion, oedema, and haemorrhage infiltration by inflammatory cells compared with the sham and ischaemia groups. Surprisingly, the observed I/R-induced increase in follicular degeneration and vascular congestion, but not of oedema, haemorrhage, and infiltration by inflammatory cells, were completely blocked by atorvastatin (Table 1).

**Table 1.** Effect of atorvastatin on ovarian histoarchitecture in ovarian I/R animal model.

	Sham	I	I/R	I + AT	IR + AT
Follicular degeneration (arbitrary unit)	0.143 ± 0.38	1.57 ± 0.54^a^	2.57 ± 0.54^ab^	0.857 ± 0.69^c^	1.29 ± 0.49^c^
Vascular Congestion (arbitrary unit)	0.43 ± 0.53	1.43 ± 0.54^a^	2.43 ± 0.79^ab^	0.71 ± 0.49^c^	1.14 ± 0.38^c^
Oedema (arbitrary unit)	0.33 ± 0.52	1.71 ± 0.49^a^	2.71 ± 0.49^ab^	1.00 ± 0.82^c^	1.43 ± 0.54^ac^
Haemorrhage (arbitrary unit)	0.57 ± 0.53	1.14 ± 0.38^a^	2.00 ± 0.58^ab^	0.57 ± 0.54^c^	1.14 ± 0.38^ac^
Infiltration by inflammatory cells (arbitrary unit)	0.29 ± 0.49	1.71 ± 0.49^a^	2.57 ± 0.53^ab^	0.71 ± 0.49^bc^	1.29 ± 0.49^ac^

Data are presented as mean ± SD of 5 replicates per group. I: Ischaemia, I/R: Ischaemia/reperfusion, AT: Atorvastatin.

^a^ *p* < 0.05 versus sham, ^b^*p* < 0.05 versus I, ^c^*p* < 0.05 versus IR, ^d^*p* < 0.05 versus I+ AT using one-way analysis of variance (ANOVA) followed by Tukey's post hoc test for pairwise comparison.

### Assessment of folliculogenesis

As shown in Table 2, a significant decrease was observed in the number of primordial, primary, and Graafian follicles in the animals that underwent ischaemia and ischaemia/reperfusion compared with their sham-operated counterparts. Interestingly, atorvastatin almost completely inhibited the observed decrease in the number of Graafian follicles, but partially ameliorated the observed reduction in the primordial and primary follicles. Furthermore, the number of secondary follicles was comparable across all groups. In addition, the number of tertiary (antral) follicles was unaltered in the ischaemia group but significantly reduced in the ischaemia/reperfusion group compared with the sham group. I/R-led reduction in tertiary (antral) follicles was significantly reversed by atorvastatin. Furthermore, a significant decrease in the number of corpus lutea was observed in ischaemia and ischaemia/reperfusion groups, which was almost completely prevented by atorvastatin treatment. Moreover, a significant increase in the number of atretic follicles was observed after ischaemia and ischaemia/reperfusion compared with the sham group. Ischaemia and I/R-induced increase in the number of Atretic follicles was significanty blocked by atorvastatin treatment.

**Table 2.** Effect of atorvastatin on ovarian folliculogenesis in ovarian I/R animal model.

	Sham	I	I/R	I + AT	IR + AT
Primordial Follicles (n)	162.00 ± 8.17	134.00 ± 8.17^a^	122.143 ± 6.90^a^	155.29 ± 7.56^bc^	149.29 ± 9.51^abc^
Primary Follicles (n)	42.00 ± 1.82	16.57 ± 1.53^a^	11.29 ± 1.49^a^	39.14 ± 1.69^bc^	36.71 ± 2.76^abc^
Secondary Follicles (n)	12.57 ± 1.53	10.71 ± 2.76	10.57 ± 2.79	11.71 ± 1.76	11.86 ± 0.69
Tertiary (antral) Follicles (n)	15.71 ± 1.49	14.00 ± 2.58	10.71 ± 0.76^a^	15.43 ± 1.53	14.286 ± 1.76
Graafian Follicles (n)	6.29 ± 0.76	4.29 ± 0.76^a^	4.00 ± 0.82^a^	5.71 ± 0.76^bc^	5.14 ± 0.69
Corpus luteum (n)	2.40 ± 0.55	1.20 ± 0.45^a^	0.80 ± 0.84^a^	2.40 ± 0.55^bc^	1.60 ± 0.54
Atretic follicles (n)	10.33 ± 0.58	42.67 ± 0.58^a^	51.67 ± 1.16^a^	23.00 ± 1.00^bc^	22.67 ± 0.58^c^

Data are presented as mean ± SD of 5 replicates per group. I: Ischaemia, I/R: Ischaemia/reperfusion, AT: Atorvastatin, n: number.

^a^ p < 0.05 versus sham, ^b^*p* < 0.05 versus I, ^c^*p* < 0.05 versus IR, ^d^*p* < 0.05 versus I+ AT using one-way analysis of variance (ANOVA) followed by Tukey's post hoc test for pairwise comparison.

### Markers of oxidative stress and activities of enzymatic antioxidants

**Figure 2.**
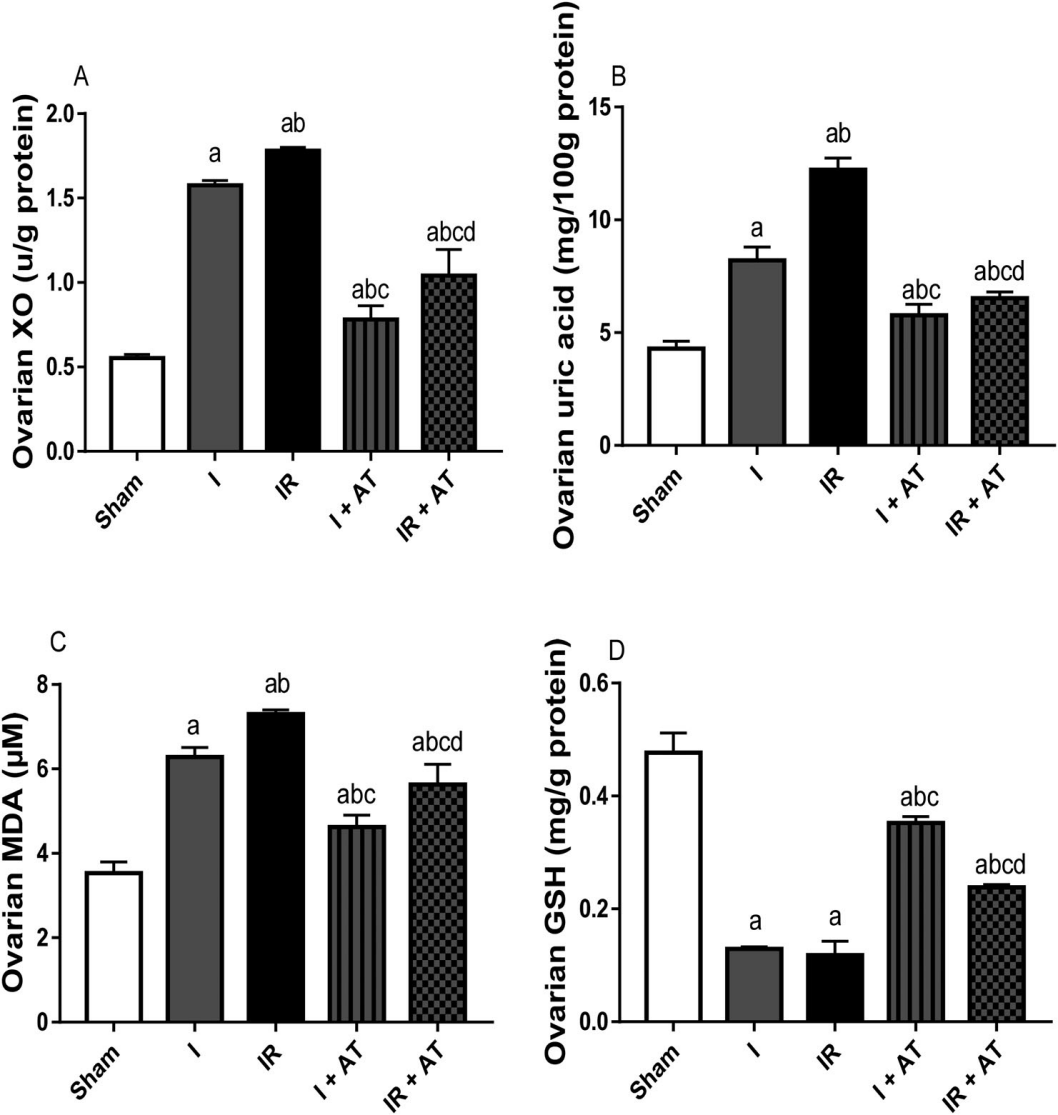
Effect of atorvastatin on markers of oxidative stress in ovarian I/R animal model. Data are presented as mean ± SD of 8 replicates per group. I: Ischaemia, I/R: Ischaemia/reperfusion, AT: Atorvastatin. ^a^ *p* < 0.05 versus sham, ^b^*p* < 0.05 versus I, ^c^*p* < 0.05 versus IR, ^d^*p* < 0.05 versus I+ AT using one-way analysis of variance (ANOVA) followed by Tukey's post hoc test for pairwise comparison.

Ovarian torsion led to a significant increase in ovarian XO, UA, and MDA, while it significantly reduced ovarian GSH concentration compared with the sham-operated rats. In addition, I/R caused marked increase in ovarian XO, UA and MDA, and significant reduction in GSH level when compared with the sham-operated and ischaemia groups. The observed ischaemia and I/R-induced rise in markers of oxidative stress were significantly alleviated by atorvastatin (Figure 2).

### Activities of enzymatic antioxidants

**Figure 3.**
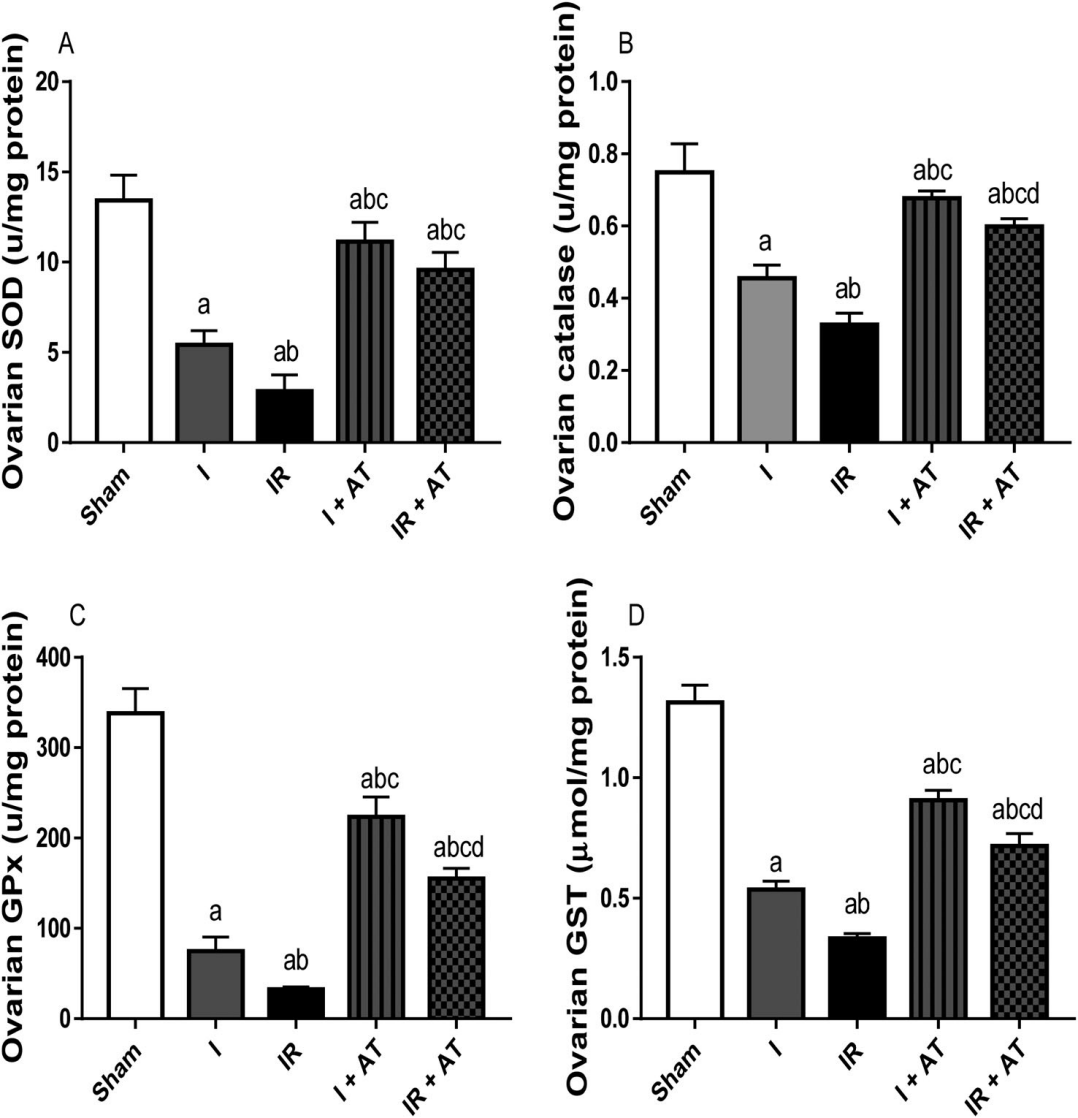
Effect of atorvastatin on the activities of enzymatic antioxidant in ovarian I/R animal model. Data are presented as mean ± SD of 8 replicates per group. I: Ischaemia, I/R: Ischaemia/reperfusion, AT: Atorvastatin. ^a^ *p* < 0.05 versus sham, ^b^*p* < 0.05 versus I, ^c^*p* < 0.05 versus IR, ^d^*p* < 0.05 versus I+ AT using one-way analysis of variance (ANOVA) followed by Tukey's post hoc test for pairwise comparison.

Ischaemia and I/R significantly reduced the activities of SOD, catalase, GPx, and GST in the ovarian tissues compared with the sham group, and sham and ischaemia groups respectively. Ischaemia and I/R-induced decline in SOD, catalase, GPx, and GST activities were significantly blunted by atorvastatin treatment (Figure 3).

### Inflammatory markers

**Figure 4.**
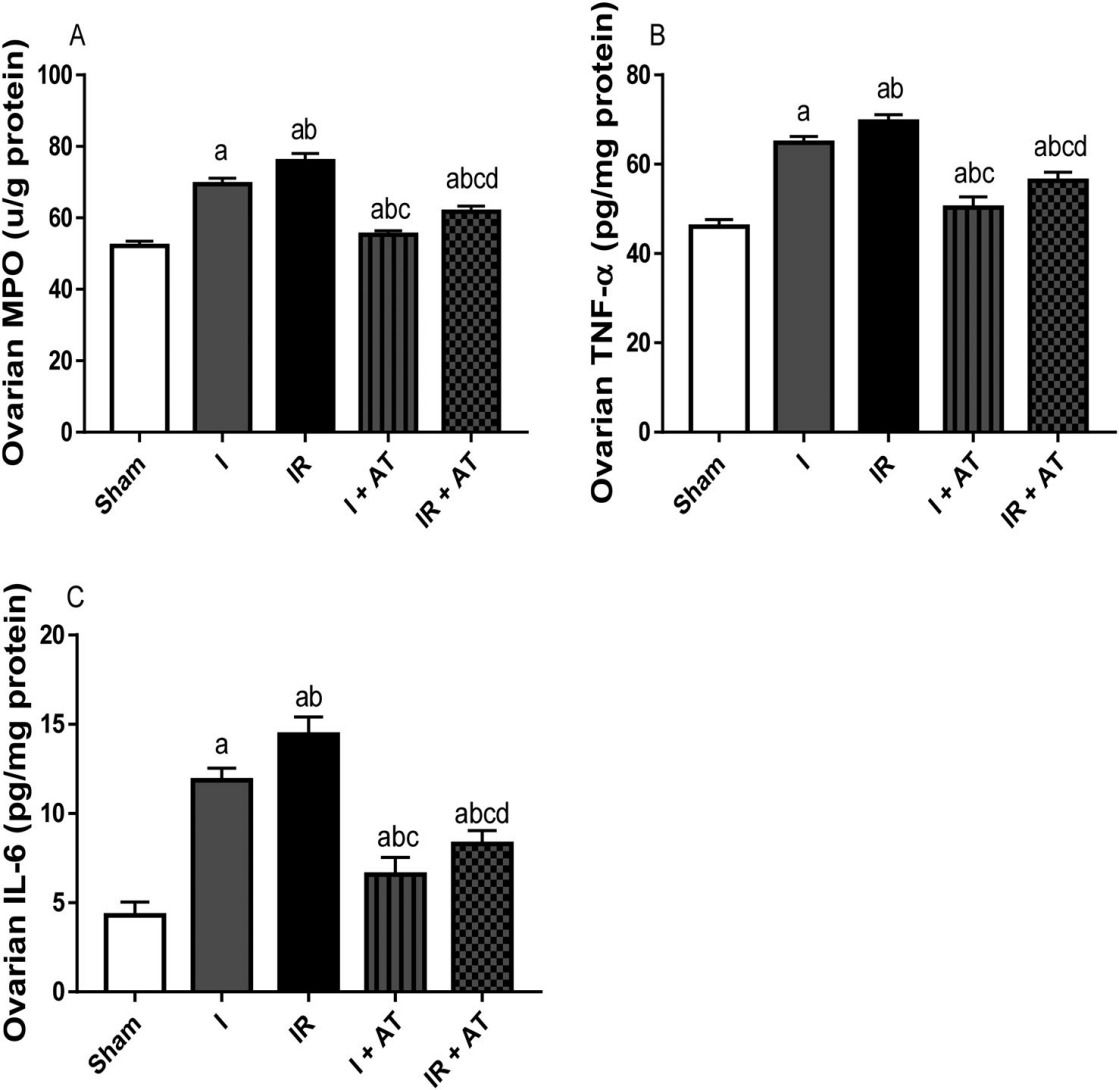
Effect of atorvastatin on inflammatory markers in ovarian I/R animal model. Data are presented as mean ± SD of 8 replicates per group. I: Ischaemia, I/R: Ischaemia/reperfusion, AT: Atorvastatin. ^a^ *p* < 0.05 versus sham, ^b^*p* < 0.05 versus I, ^c^*p* < 0.05 versus IR, ^d^*p* < 0.05 versus I+ AT using one-way analysis of variance (ANOVA) followed by Tukey's post hoc test for pairwise comparison.

Ischaemia led to a significant increase in MPO activity and TNF-α, and IL-6 concentrations in the ovaries when compared with the sham-operated animals. Ischaemia/reperfusion also caused a significant increase in these inflammatory markers compared with the sham and ischaemia groups. These alterations were significantly mitigated by atorvastatin (Figure 4).

### Genotoxicity and apoptotic markers

**Figure 5.**
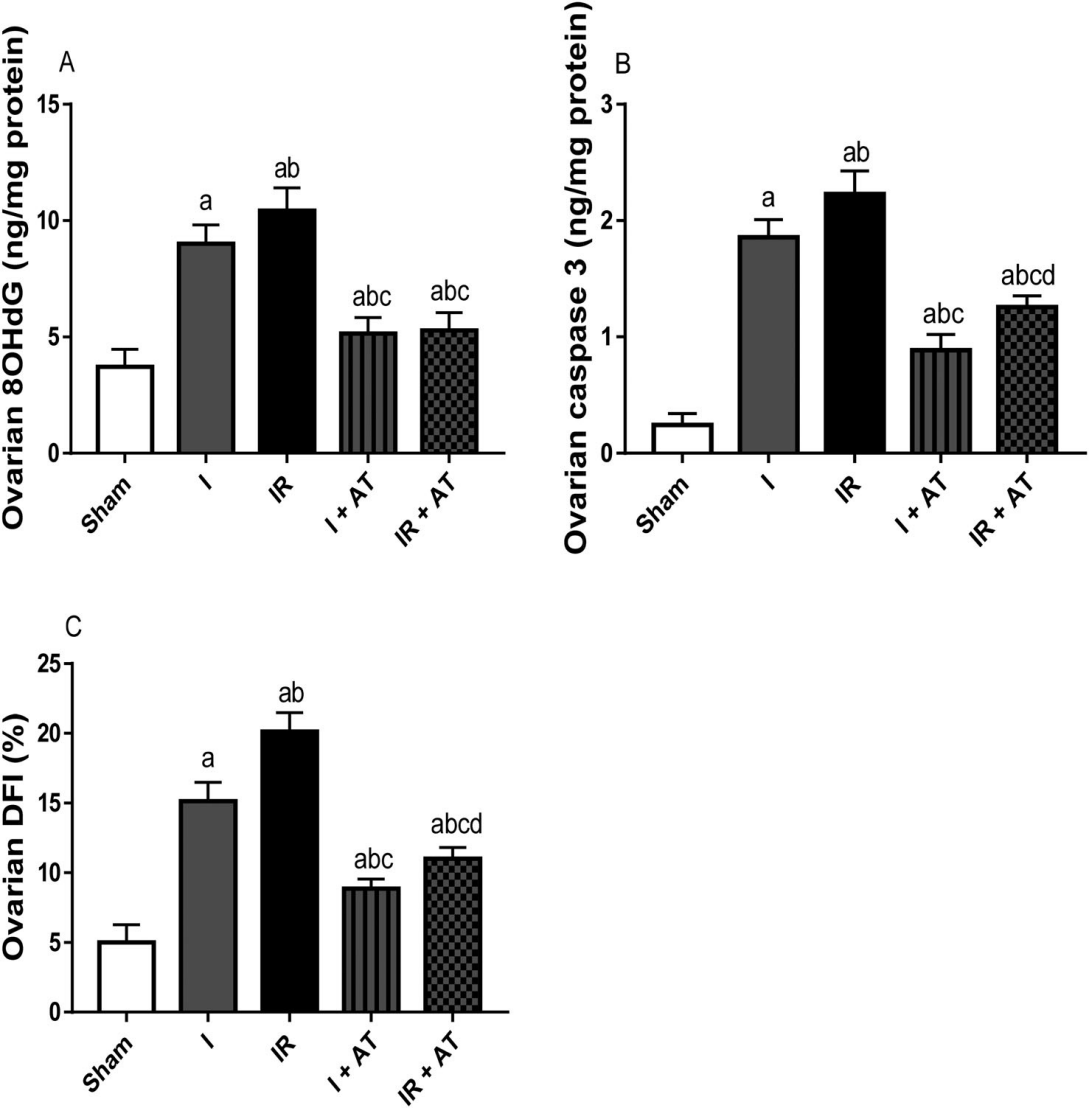
Effect of atorvastatin on markers of genotoxicity and apoptosis in ovarian I/R animal model. Data are presented as mean ± SD of 8 replicates per group. I: Ischaemia, I/R: Ischaemia/reperfusion, AT: Atorvastatin. ^a^ *p* < 0.05 versus sham, ^b^*p* < 0.05 versus I, ^c^*p* < 0.05 versus IR, ^d^*p* < 0.05 versus I+ AT using one-way analysis of variance (ANOVA) followed by Tukey's post hoc test for pairwise comparison.

Ischaemia following ovarian torsion led to a significant rise in 8OHdG level (marker of oxidative DNA damage and genotoxicty), caspase 3 activity (marker of apoptosis), and DFI (marker of apoptosis) when compared with the sham group. Also, I/R significantly increased these genotoxicity and apoptotic markers in the ischaemia/reperfusion group compared with the sham and ischaemia groups. The observed ischaemia and I/R-driven increase in genotoxicity and apoptosis was markedly suppressed by atorvastatin (Figure 5).

### Marker of cell adhesion

**Figure 6.**
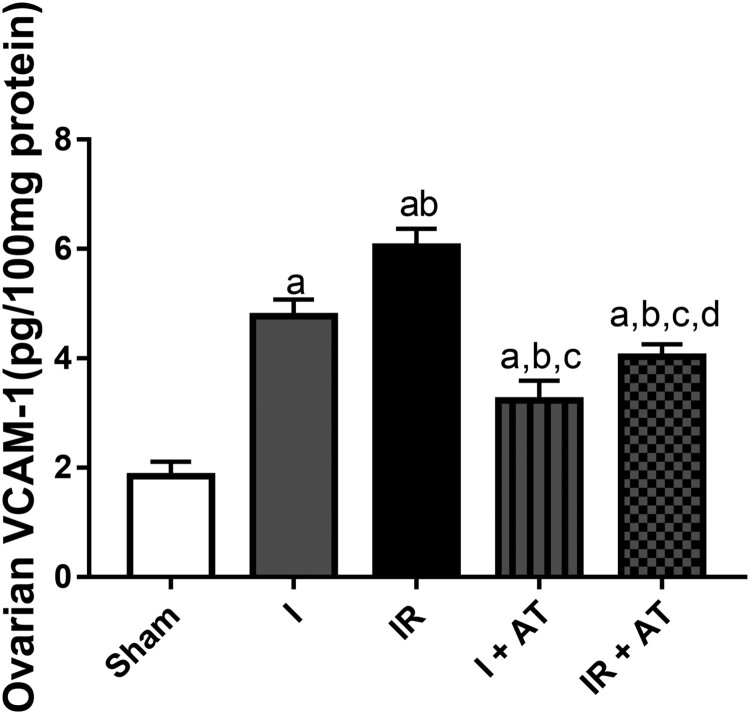
Effect of atorvastatin on vascular cell adhesion molecule (VCAM) in ovarian I/R animal model. Data are presented as mean ± SD of 8 replicates per group. I: Ischaemia, I/R: Ischaemia/reperfusion, AT: Atorvastatin. ^a^ *p* < 0.05 versus sham, ^b^*p* < 0.05 versus I, ^c^*p* < 0.05 versus IR, ^d^*p* < 0.05 versus I+ AT using one-way analysis of variance (ANOVA) followed by Tukey's post hoc test for pairwise comparison.

Ischaemia and ischaemia/reperfusion led to a significant increase in ovarian VCAM-1 compared with the sham group. In addition, the observed increase in VACM-1 in the ischaemia/reperfusion group was markedly more than that in ischaemia group. Also, atorvastatin significantly repressed ischaemia and I/R-induced upregulation of ovarian VCAM-1 (Figure 6).

## Discussion

The study demonstrates that HMG-CoA reductase inhibition by atorvastatin attenuates I/R-induced distortion of ovarian structural integrity and function in ovarian T/D animal model. In previous studies, I/R injury has been shown to increase ROS generation and enhance lipid peroxidation in the ovaries [31, 32]. Nevertheless, data from the present study revealed for the first time that ovarian I/R-induced lipid peroxidation is consequent to, at least in part, elevated XO activity, UA levels, VCAM-1 expression, and caspase 3 activity. Also, atorvastatin militates against oxido-inflammatory damage and apoptosis by downregulating VCAM-1 expression and XO/UA/caspase 3 signaling, which mediates its protective activity against I/R ovarian injury.

Accumulation of excessive levels of UA is an integral player and a major source of intracellular oxidative stress. UA is generated as the end product of purine nucleic acids, adenine and guanine metabolism. Adenosine monophosphate is converted to inosine via deamination and dephosphorylation, while guanine monophosphate is converted to guanosine by nucleotidase [33]. These nucleosides, inosine and guanosine, are broken down into hypoxanthine and guanine respectively [28, 33]. Hypoxanthine is oxidized, while guanine is deaminated to form xanthine, which is in turn oxidized by xanthine oxidase to form UA [28]. Although UA exerts antioxidant activity by chelating metal ions such as iron and copper and converting them to their poorly active forms [34], it forms free radicals when it interacts with peroxynitrite [35, 36], and oxidized lipids [37]. Studies have reported compelling evidence that implicate UA as a trigger of oxidative stress, inflammation, and apoptosis [38–40]. Our findings in this study indicate that IR-induced increased MDA and reduced GSH may have resulted from I/R – led elevated XO activity and UA levels. This study confirms that elevated UA and the subsequent oxidative stress play an important role in ovarian I/R injury. Hence, blockade of XO/UA signaling might be a potent strategy for averting I/R-induced oxidative stress.

Since cells and tissues protect themselves from oxidative injury by the scavenging activity of endogenous antioxidants [14], the observed I/R-led rise in XO/UA may account for the decline in the activities of enzymatic antioxidants (SOD, catalase, GPx and GST) and GSH. I/R-driven upregulation of XO/UA may also explain the negative impacts of T/D on ovarian histoarchitecture, follicles, and folliculogenesis. The high content of polyunsaturated fatty acids in the ovarian follicles [41] predisposes them to ROS attack. IR-induced elevated UA promotes lipid peroxidation and disruption of the structural integrity of the ovarian plasma membrane [41]. This exposes the follicles and other cellular organelles to oxidative damage [42–44], especially oxidative DNA damage [29] evidenced in this study by the rise in 8OHdG. In the current study, I/R caused an increase in the ovarian concentrations of MDA and 8OHdG, which was associated with reduced activities of SOD, catalase, GPx and GST and GSH level.

In addition, ovarian I/R did not only induce ovarian injury evidenced by distortion of the ovarian histoarchitecture and prominent vascular congestion, oedema, hemorrhage, and infiltration by inflammatory cells, it also led to increased follicular degeneration evidenced by marked reduction in follicular counts and increased number of atretic follicles. These alterations were significantly blunted by atorvastatin. Based on these observations, it is safe to infer that HMG-CoA reductase inhibition by atorvastatin may play a novel role in alleviating I/R-induced ovarian injury by suppressing XO/UA signaling and maintaining a positive redox state.

Another striking finding of this study is the blockade of I/R-induced elevation of VCAM-1 by atorvastatin. VCAM-1 is a glycoprotein that is activated by ROS and pro-inflammatory cytokines such as TNF-α [45], and regulates inflammation-dependent vascular adhesion and transendothelial migration of leukocytes [46]. Activation of cytokines facilitates binding of ligands to VCAM-1 and initiates the activation of calcium fluxes and Rac1 [45, 47], which in turn induce the downstream activation of nicotinamide adenine dinucleotide phosphate (NADPH) oxidase 2, resulting in ROS generation [47]. Thus, upregulation of VCAM-1 expression in the present study could be due to I/R-induced ROS generation or I/R-induced activation of TNF-α and IL-6. Also, the observed I/R-led rise in VCAM-1 expression may explain the increased activity of MPO in the ovarian tissue. It is likely that I/R-mediated activation of TNF-α promotes the binding of this cytokine with TNF receptor (TNFR) and consequent TNFR homotrimerization and recruitment of adaptor proteins to the intracellular domain, leading to inflammation, oxidative stress, and apoptosis [48, 49]. The pleiotropic biological activities of TNF-α and VCMA-1 may promote an oxido-inflammatory response, which possibly triggered DNA fragmentation and apoptosis of the ovarian tissue [29] evidenced by a rise in ovarian DFI and caspase 3 activity. Astonishingly, atorvastatin ameliorated I/R-induced elevated VCAM-1 and associated rise in TNF-α, IL-6 and MPO activity. Just like simvastatin, it is likely that atorvastatin inhibited the expression of pro-inflammatory cytokines [50], thus prevent the activation of TNF-α and the binding of TNF-α with TNFR, and consequently IR-driven oxidative stress, inflammation, and caspase 3-mediated apoptosis.

In summary, this study provides the first confirmation of the key role of VCAM-1 and XO/UA signaling in ovarian I/R injury. Furthermore, the results presented in this study reveal that atorvastatin-mediated downregulation of VCAM-1 and XO/UA/caspase 3 signaling averts oxidative damage, inflammation, and apoptosis induced by ovarian ischaemia/reperfusion injury. These findings identify VCAM-1 and XO/UA/caspase 3 as critical signaling pathways in ovarian I/R injury and unravel a novel therapeutic target for ovarian I/R injury.

## Data Availability

Data is available upon reasonable request.
